# Binding affinity of amyloid oligomers to cellular membranes is a generic indicator of cellular dysfunction in protein misfolding diseases

**DOI:** 10.1038/srep32721

**Published:** 2016-09-13

**Authors:** Elisa Evangelisti, Roberta Cascella, Matteo Becatti, Giovanna Marrazza, Christopher M. Dobson, Fabrizio Chiti, Massimo Stefani, Cristina Cecchi

**Affiliations:** 1 Department of Experimental and Clinical Biomedical Sciences “Mario Serio” and Research Centre on the Molecular Basis of Neurodegeneration (CIMN), University of Florence, Viale Morgagni 50, 50134 Florence, Italy; 2 Department of Chemistry “Ugo Schiff”, University of Florence, Via della Lastruccia 3, 50019 Florence, Italy; 3 Department of Chemistry, University of Cambridge, Lensfield Road, CB2 1EW Cambridge, UK

## Abstract

The conversion of peptides or proteins from their soluble native states into intractable amyloid deposits is associated with a wide range of human disorders. Misfolded protein oligomers formed during the process of aggregation have been identified as the primary pathogenic agents in many such conditions. Here, we show the existence of a quantitative relationship between the degree of binding to neuronal cells of different types of oligomers formed from a model protein, HypF-N, and the GM1 content of the plasma membranes. In addition, remarkably similar behavior is observed for oligomers of the Aβ_42_ peptide associated with Alzheimer’s disease. Further analysis has revealed the existence of a linear correlation between the level of the influx of Ca^2+^ across neuronal membranes that triggers cellular damage, and the fraction of oligomeric species bound to the membrane. Our findings indicate that the susceptibility of neuronal cells to different types of misfolded oligomeric assemblies is directly related to the extent of binding of such oligomers to the cellular membrane.

Aberrant protein oligomers are conformationally heterogeneous assemblies associated with many protein deposition disorders. They are formed early in the aggregation path of peptides and proteins^1^, or are released from mature fibril ends^2^ or are generated by secondary processes involving mature fibrils^3^. Many such species have been shown to be pathogenic, a characteristic that appears to result from their aberrant interactions with cellular membranes, impairing the ability of the latter to maintain cellular homeostasis^4^,^5^,^6^. An increasing body of data indicates that oligomers of a given peptide or protein that are formed under different conditions, or over different periods of time, can possess quite different morphological and structural features that are reflected in different degrees of toxicity^1^,^7^,^8^,^9^,^10^,^11^,^12^,^13^,^14^.

Particularly detailed studies of this phenomenon have involved the N-terminal domain of *E.coli* HypF (HypF-N), a model protein that aggregates under different conditions into two well-defined and highly stable forms of oligomeric species, denoted type A and type B^10^. The two types of HypF-N oligomers have been shown to have similar sizes and morphologies, but to display very different toxicities when added to the extracellular medium of cultured SH-SY5Y cells, perfused in rat hippocampal slices or injected in rat brains, a finding attributed to the different solvent-exposure and flexibility of hydrophobic regions of the protein^15^,^16^,^17^. It has also been reported that the amyloid β peptide 42 (Aβ_42_) peptide, whose aggregation is directly linked to the onset and progression of Alzheimer’s disease, forms two types of oligomers under slightly different conditions that are of similar size but possessing very different physical and biological properties^12^,^14^. These oligomers, denoted A+ and A−, have been found to be toxic and benign, respectively, to PC12 rat adrenal medulla cells and rat primary cortical neuronal cells, an observation again attributed to the higher solvent-exposure of hydrophobic regions in the toxic A+ oligomers^12^. Other forms of Aβ_42_ oligomers, found in human brains and denoted type 1 and type 2, have also been found to display markedly different toxicities to neurons^14^.

The ability of aberrant protein oligomers to penetrate and disrupt cellular membranes and induce toxicity is likely to result from direct interactions with the lipid bilayers^6^,^18^,^19^. The ability of cell membranes to bind oligomeric aggregates appears to depend in turn on the physicochemical properties of both the oligomers and the membranes, which for the latter are determined in large part by their lipid composition^20^. In particular, the monosialotetrahexosylganglioside GM1 has been found to be an important factor in the context of Alzheimer’s disease^21^. GM1, together with cholesterol and sphingomyelin, is abundant in lipid rafts, domains within the cell membrane that contain a vast array of membrane proteins including channels and receptors^22^. Indeed, neuronal membranes are highly enriched in lipid rafts, and perturbations within these membrane regions have been associated with neuronal dysfunction and neurodegenerative conditions. In particular, disruption of lipid rafts appears to protect mature neurons against oligomer cytotoxicity^21^ and alterations in the membrane distribution of GM1 and GM2 have recently been reported in the brains of patients suffering from Alzheimer’s disease^23^. Moreover, the GM1 content has been found to increase both with age^24^ and in the brains of Alzheimer’s disease patients compared to healthy controls^23^. The importance of GM1 in promoting amyloid aggregation and in modulating the interaction of aggregates with cell membranes and its associated role in mediating aggregate cytotoxicity, have been explored in some detail^24^,^25^. It is not clear, however, whether a quantitative relationship exists between GM1 abundance on cell surfaces and the ability of the corresponding membranes to recruit protein oligomers and consequently impair cell viability.

In the present study, we have investigated the origin of these observations by modifying in a reproducible and quantitatively defined manner the content of GM1 in the plasma membrane of human SH-SY5Y neuroblastoma cells by more than an order of magnitude, from 26.2 ± 5.3% to 352 ± 22% with respect to the basal GM1 level, taken as 100%. Then, using the type A and type B oligomers formed from the HypF-N protein and the A+ and A− oligomers of the Aβ_42_ peptide, we have examined the effects of the GM1 abundance on the binding of the oligomers to neuronal membranes, the impairment of cellular homeostasis and the overall viability of the cells. Experimental protocols commonly used to study amyloid oligomeric species are effective in the nM-μM range concentration, and here we used 6–12 μM concentration of HypF-N as this was optimised in previous experiments as the ideal concentration to observe calcium influx and the resulting toxicity^7^,^10^. Accordingly, we used 10 μM concentrations of Aβ oligomers, as previous studies of soluble Aβ species in an Alzheimer’s disease brain tissue have shown a wide range of values (0.021 ± 0.089 μg/g to 0.33 ± 0.49 μg/g of tissue)^26^. Our results show that the effects of the different types of oligomers on the cells have different dependences on the membrane GM1 content. On the basis of these data, we have derived expressions that rationalize these effects in terms of the binding affinities for the membrane of the different species. This analysis shows that their biological effects are directly related to the quantity of oligomers bound to the membrane regardless of their sequence or structure, with their effects becoming similar when similar amounts of different oligomers are bound to the membrane.

## Results

### The binding affinity of HypF-N oligomers to cellular membranes correlates with the GM1 content

In order to monitor the GM1 content in neuronal membranes we employed double label confocal microscopy using anti-GM1 antibodies and the cholera toxin subunit B (CTX-B), and also flow cytometric analysis in conjunction with the CTX-B conjugate. CTX-B, a component of a heat-labile enterotoxin produced by *Vibrio cholerae*, is a probe commonly used to detect GM1 ganglioside. Increases in GM1 levels were achieved by incubating SH-SY5Y neuroblastoma cells with 10, 15, 20, 50 or 100 μg/ml GM1 from bovine brain; the highest concentration resulted in an increase in the GM1 level by more than a factor of 3, up to 352 ± 22% with respect to the basal GM1 level (Fig. S1). Decreases in GM1 levels were achieved following incubation of the cells for 48 h in solutions containing 0.5, 1.0, 10 or 25 μM D-threo-1-phenyl-2-decanoylamino-3-morpholino-1-propanol (PDMP), a glucosylceramide synthase inhibitor that blocks the natural synthesis of GM1^27^; at the highest concentration of PDMP the GM1 level was reduced to 26.2 ± 5.3% of the basal level (Fig. S1).

**Figure 1:**
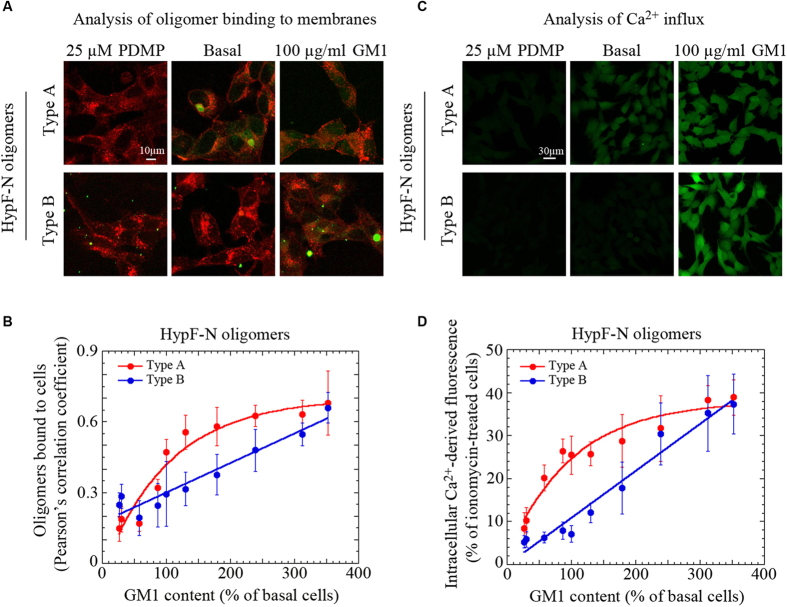
Binding of HypF-N oligomers to cells with differing GM1 contents and the ensuing Ca^2+^ influx. (**A**) Representative confocal scanning microscopy images of GM1-depleted (25 μM PDMP), basal and GM1-enriched (100 μg/ml GM1) cells treated for 1 h with type A or type B HypF-N oligomers (12 μM, monomer equivalents). Red and green fluorescence indicates the cell membranes and the HypF-N oligomers (small dots), respectively. Large aggregates (big dots) result from oligomer clustering on the cell membrane. (**B**) Plot showing the extent of oligomer binding to the membrane against GM1 content, for type A (red) or type B (blue) HypF-N oligomers (12 μM, monomer equivalents). Red and blue lines represent the best fits to hyperbolic and linear functions, respectively. Variable numbers of cells (12–22) in three different experiments were analysed for each condition. Error bars refer to standard deviation (S.D.) (**C**) Representative confocal scanning microscopy images of GM1-depleted (25 μM PDMP), basal and GM1-enriched (100 μg/ml GM1) cells showing levels of intracellular free Ca^2+^ following treatment for 1 h with type A or type B oligomers of HypF-N (12 μM, monomer equivalents); the green fluorescence arises from Ca^2+^ binding to the intracellular Fluo3-AM probe. (**D**) Intracellular Ca^2+^ fluorescence against GM1 content for type A (red) or type B (blue) HypF-N oligomers. Other details are as for panel B.

We then investigated the degrees of co-localization of the two different types of HypF-N oligomers (green channel) with cell membranes (red channel) using scanning confocal microscopy in cells with basal GM1 content (Fig. 1A). The degree of co-localization, as assessed from the Pearson’s correlation coefficient (PCC), was found to be 0.47 ± 0.05 for type A oligomers (small dots) and 0.29 ± 0.14 for type B oligomers (small dots) when a 12 μM concentration of aggregates (in monomer equivalents) were added to the cell media (Fig. 1A). In cells treated with 100 μg/ml GM1, and containing the highest concentration of GM1, the PCC values were 0.68 ± 0.13 and 0.66 ± 0.06 for type A and type B oligomers, respectively, indicating that GM1-enriched cells displayed a higher ability to recruit either type of oligomer (Fig. 1A,B). By contrast, the ability of GM1-depleted cells, treated with 25 μM PDMP, to recruit type A or type B oligomers was greatly reduced (PCC = 0.15 ± 0.05) or unchanged (PCC = 0.25 ± 0.04), respectively, compared to cells with basal GM1 content (Fig. 1A,B). Confocal microscopy images with all levels of GM1 are reported in the Fig. S2A.

A plot of the PCC *versus* the GM1 content in cells treated with type A oligomers fitted closely to a hyperbolic function, whereas the data points for the type B oligomers are almost linearly correlated with the GM1 content (Fig. 1B). Nevertheless, the values at high and low GM1 levels are similar, indicating that HypF-N type A oligomer binding to the cell membrane occurs with high affinity and apparent saturation kinetics, whereas the GM1-dependence of HypF-N type B oligomer binding follows linear kinetics and displays low affinity. It is possible that the linear dependence observed between PCC and the GM1 content results from such a low affinity and is indeed the first part of a hyperbolic dependence whose plateau cannot be observed due to the difficulty of reaching GM1 concentrations higher than those reached here. These findings show that the ability of the membrane to recruit oligomers to the cell surface depend primarily on the lipid composition of the membrane.

### The dysregulation of cytosolic Ca^2+^ induced by HypF-N oligomers correlates with the GM1 content

Many studies have revealed that the influx of Ca^2+^ from the extracellular medium into the cytosol is an early event in the cascade of biochemical changes underlying the cytotoxicity induced by misfolded proteinaceous aggregates^28^,^29^,^30^,^31^,^32^, including HypF-N oligomers^10^,^15^. We therefore compared the effects of the two types of HypF-N oligomers in GM1-modulated cells by measuring the levels of intracellular Ca^2+^ using Fluo3-AM as a probe in conjunction with confocal microscopy. In cells with basal levels of GM1, type A and type B oligomers caused very different degrees of Ca^2+^ influx when the same concentration of aggregates (in monomer equivalents) was added to the cell medium (Fig. 1C). The Ca^2+^ influx, quantified as the percentage of the maximum value measured in cells exposed for 1 h to 1.0 μM ionomycin (Fig. S2C), a well known ionophore that permeabilizes the cell membrane to Ca^2+^, was found to be 25.5 ± 4.4% and 7.1 ± 1.9% for a 12 μM concentration of type A and type B oligomers, respectively (Fig. 1D). In cells with the lowest GM1 levels, exposure to the two types of oligomers led to small and similar intracellular concentrations of Ca^2+^, 8.5 ± 3.6% and 5.3 ± 1.4% of the maximum value obtained with ionomycin for type A and B oligomers, respectively (Fig. 1C,D). In cells with the highest GM1 levels, the influx of cytosolic Ca^2+^ was high but again very similar, 38.9 ± 4.1% and 37.3 ± 6.9% of the maximum value measured with ionomycin, for type A and type B oligomers, respectively (Fig. 1C,D). Confocal microscopy images showing Ca^2+^ influx for both oligomer types and with all levels of GM1 are reported in the Fig. S2B. The plot of the fluorescence associated with the influx of intracellular Ca^2+^ against the GM1 content fitted well to a hyperbolic function for cells treated with type A oligomers (Fig. 1D). By contrast, as found with the colocalization data in Fig. 1B, the same plot was essentially linear for cells treated with type B oligomers (Fig. 1D). Cytosolic Ca^2+^ levels were not observed to change significantly in cells with varying GM1 levels in the absence of oligomers, suggesting that the variation in GM1 content does not by itself perturb Ca^2+^ homeostasis (Fig. S3A,C).

We next sought to assess whether or not the membrane GM1 content could also modulate the effect of different types of HypF-N oligomers on the bilayer permeability and viability of cultured SH-SY5Y cells. The degree of membrane permeability was measured by means of confocal microscopy by monitoring the release of intracellular calcein from cells that had been loaded with the fluorescent probe calcein-AM^33^, and cell viability was measured with the MTT reduction assay. The results obtained with both probes were similar to those obtained by monitoring the intracellular Ca^2+^: similar hyperbolic and linear curves were obtained in plots of intracellular calcein fluorescence or MTT reduction *versus* GM1 content in cells exposed to type A or type B oligomers, respectively (Fig. S4). Calcein levels were not observed to change significantly in cells with varying GM1 levels in the absence of oligomers, suggesting that the variation in GM1 content does not by itself perturb calcein release (Fig. S3B,D). These findings confirm that the observed cytotoxicity is closely related to the affinity of the oligomers for the membrane.

### Ca^2+^ dysregulation depends on the binding affinity of oligomers to cellular membranes

**Figure 2:**
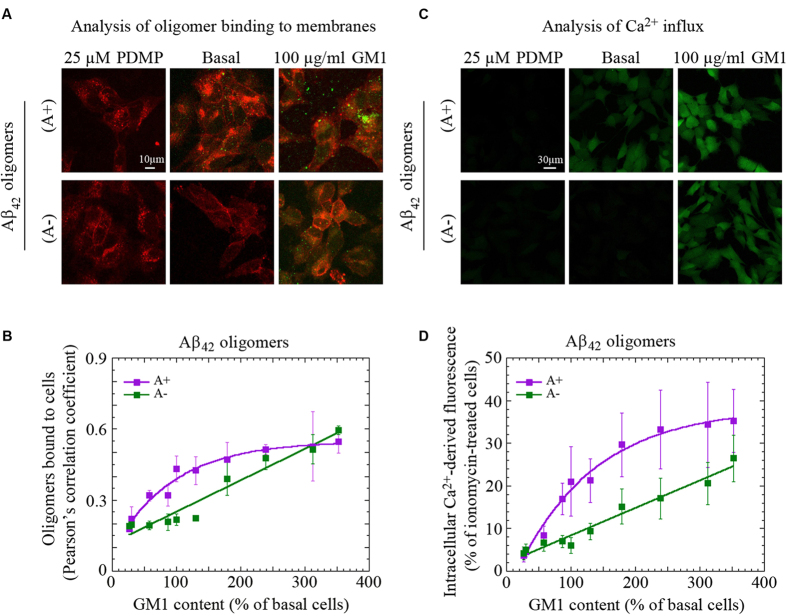
Binding of Aβ_42_ oligomers to cells with differing GM1 content and the ensuing Ca^2+^ influx. (**A**) Representative confocal scanning microscopy images of GM1-depleted (25 μM PDMP), basal and GM1-enriched (100 μg/ml GM1) cells treated for 1 h with A+ or A− Aβ_42_ oligomers (10 μM, monomer equivalents). Red and green fluorescence emission is from cell membranes and Aβ_42_ oligomers, respectively. (**B**) Plot showing the extent of oligomer binding to the cell membrane against GM1 content, for A+ (violet) or A− (green) Aβ_42_ oligomers (10 μM, monomer equivalents). Violet and green lines represent the best fits to hyperbolic and linear functions, respectively. Variable numbers of cells (12–22) in three different experiments were analysed for each condition. Error bars refer to S.D. (**C**) Representative confocal scanning microscopy images of GM1-depleted (25 μM PDMP), basal and GM1-enriched (100 μg/ml GM1) cells showing the levels of intracellular free Ca^2+^ following exposure for 1 h to A+ or A− oligomers of Aβ_42_ (10 μM, monomer equivalents); the green fluorescence arises from Ca^2+^ binding to the intracellular Fluo3-AM probe. (**D**) Intracellular Ca^2+^ fluorescence against GM1 content for A+ (violet) or A− (green) Aβ_42_ oligomers. Other details are as for panel B.

We then investigated in a completely analogous fashion the behavior of the two different types of oligomers (A+ and A−) formed from the Aβ_42_ peptide^12^. The results obtained on membrane binding and Ca^2+^ influx with the two types of Aβ_42_ oligomers were found to be very similar to those obtained with the two types of HypF-N oligomers (Figs 2 and S5), suggesting that the phenomenon observed in these experiments is generic rather than specific to a given type of oligomeric aggregate.

In addition, to study the contribution of endocytotic, sorting and degradation processes that could interfere with the analysis of the oligomer binding to the cellular membrane, we treated basal and GM1-enriched cells with A+ Aβ_42_ oligomers for 15 min at 37 °C, for 60 min at 37 °C or for 60 min at 16 °C. The amount of oligomers bound to the cellular membrane appears to be similar in all experimental conditions (Fig. S6A,B), thus suggesting a very few contribution, if any, of cellular processes such as endocytosis in our experimental conditions. Moreover, no intracellular oligomers appear in the median planes of cells with increased GM1 content treated for 1 h with A+ or A− Aβ_42_ (Fig. S6C), validating the quantitative analysis of the oligomers bound to the cellular membrane in our experimental conditions.

**Figure 3:**
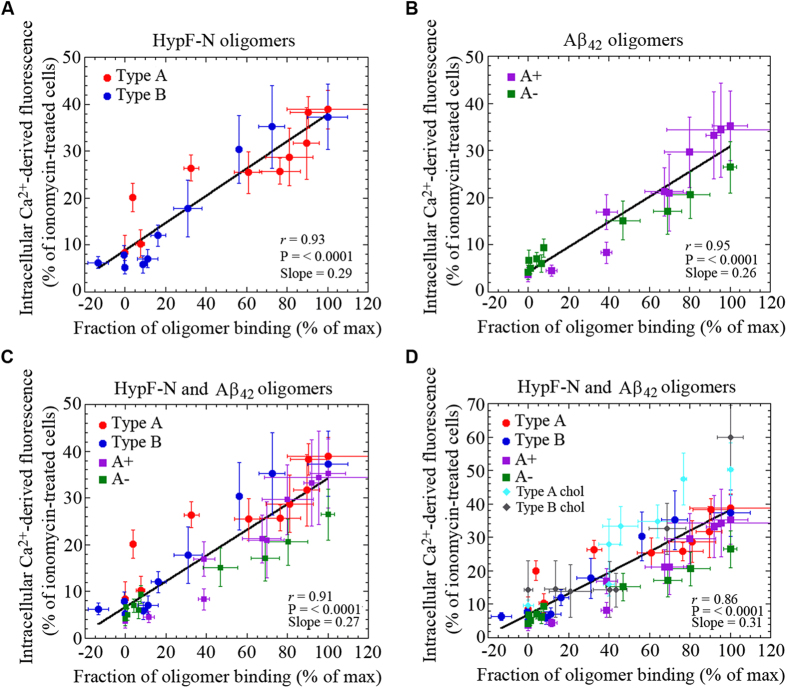
Dependence of intracellular Ca^2+^ levels on the binding affinities of HypF-N and Aβ_42_ oligomers to cells with different GM1 and cholesterol content. (**A**,**B**) Changes of the intracellular Ca^2+^ levels plotted against the fraction of oligomer binding to the plasma membrane in GM1-modulated SH-SY5Y neuroblastoma cells treated for 1 h with (**A**) 12 μM type A (red) or type B (blue) oligomers of HypF-N; (**B**) 10 μM A+ (violet) or A− (green) oligomers of Aβ_42_; (**C**) plot showing results with both pairs of HypF-N and Aβ_42_ oligomers. (**D**) Same plot as (**C**), also containing previously published data obtained with 12 μM type A (cyan) or type B (gray) HypF-N oligomers in cholesterol-modulated cells^20^.

We therefore hypothesized that the biological effects of different types of oligomers from different peptides and proteins could depend primarily on their ability to bind to the cell membrane rather than on their sequence or structure; indeed, the effects of the different oligomers become similar under conditions where similar quantities of the oligomers are bound to the membrane. In order to examine this effect further, the change of the intracellular Ca^2+^ levels was plotted against the fraction of the oligomer population bound to the plasma membrane using the data obtained from the two pairs of oligomers (Fig. 3A,B). We found a highly significant correlation for the oligomers generated from both HypF-N (r = 0.93, *P* < 0.0001) and Aβ_42_ (r = 0.95, *P* < 0.0001). The data points in both cases fit closely to a linear function, indicating that the oligomer-induced increase in free intracellular Ca^2+^ is directly proportional to the fraction of the oligomer population that is bound to the cell surface, regardless of the type of oligomer involved (compare Fig. 3A with 3B). Moreover, the close similarity of the two plots obtained with the two protein systems suggests the existence of essentially identical effects on Ca^2+^ homeostasis following binding of each type of oligomer. In fact, all the data points from the four types of oligomers fit extremely well to a linear function (Fig. 3C, r = 0.91, *P* < 0.0001), suggesting a common or generic phenomenon independent of the sequence of the polypeptide or the structure of the oligomers involved.

In order to test whether or not the biological effects of the various types of oligomers depend significantly on their ability to bind to cellular membranes, regardless of the specific lipid content, we have also examined previous experimental data relating to the effects of the different types of HypF-N oligomers on SH-SY5Y cells containing different levels of cholesterol in the plasma membrane^20^. When the intracellular Ca^2+^ levels were plotted against the fraction of oligomers bound to the membrane, all the values determined from the previous and the present data again are highly correlated (Fig. 3D, r = 0.86, *P* < 0.0001).

### Different concentrations of HypF-N oligomers result in different levels of Ca^2+^ dysregulation

**Figure 4:**
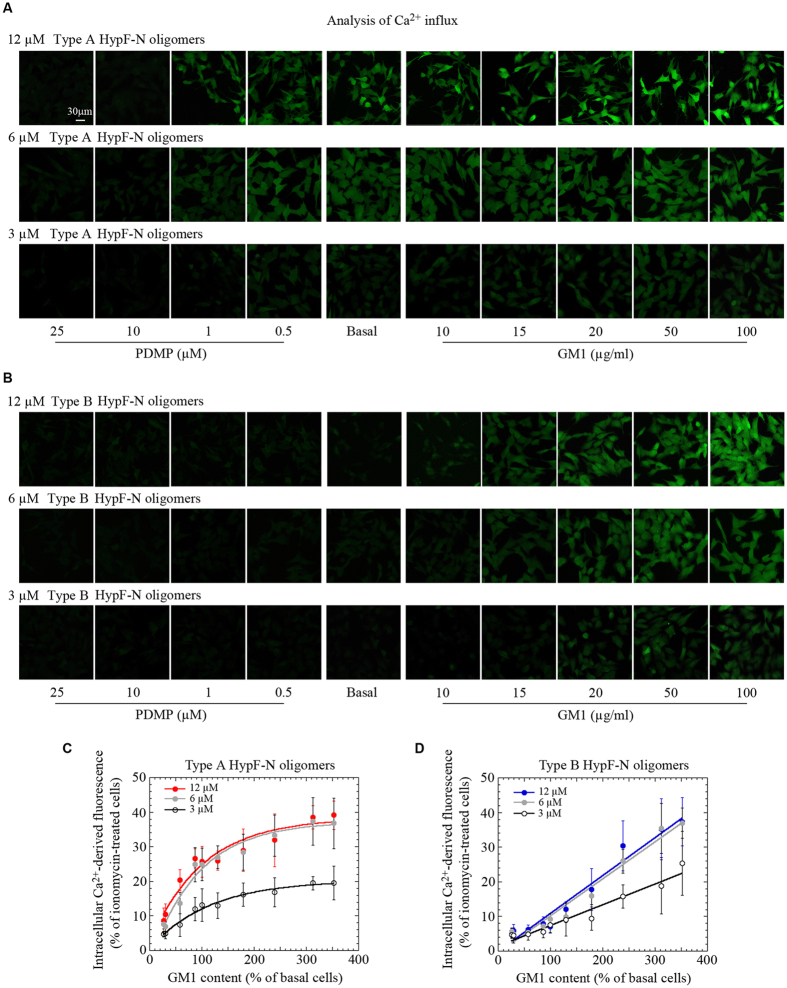
Ca^2+^ influx in cells with different GM1 content exposed to different concentrations of HypF-N oligomers. (**A**,**B**) Representative confocal scanning microscopy images of basal, GM1-enriched (GM1) and GM1-depleted (PDMP) cells showing levels of intracellular free Ca^2+^ following treatment for 1 h with type A (**A**) or type B (**B**) oligomers at 12 μM (upper images), 6.0 μM (middle images) and 3.0 μM (lower images) monomer equivalent. In all images, the green fluorescence arises from Ca^2+^ binding to the intracellular Fluo3-AM probe. (**C**,**D**) Plots showing the fluorescence associated with intracellular Ca^2+^ *versus* GM1 content after treatment of the cells with HypF-N type A (**C**) or type B (**D**) oligomers at 12 μM (red or blue lines), 6.0 μM (gray line and filled circles) or 3.0 μM (black line, empty circles) monomer equivalents. The corresponding lines represent the best fits to hyperbolic functions. Intracellular Ca^2+^ -derived fluorescences were expressed as fractional changes of ionomycin-treated cells (taken as 100%). A variable number of cells ranging from 10 to 22 were analysed for every experimental conditions in three different experiments. Error bars refer to S.D.

In order to gain mechanistic insight on the binding of type A and type B HypF-N oligomers to the cell membrane, we designed and carried out a number of experiments. We first assessed if varying the GM1 content could modulate the Ca^2+^-influx in cells exposed to lower concentrations of HypF-N oligomers. The plot of the fluorescence associated with the influx of intracellular Ca^2+^ against the GM1 content was observed to fit well to a hyperbolic function for cells treated with 3.0 μM and 6.0 μM type A oligomers, and the effects were found to be increased by a factor of two at the higher concentration (Fig. 4A,C). In addition, the plot of the intracellular Ca^2+^-derived fluorescence of cells treated with 3.0 μM or 6.0 μM concentration of type B oligomers against the GM1 content was found to be linear; a similar linear relationship was found in both cases, albeit with a decreased slope at the lower concentration (Fig. 4B,D). The comparison between the two types of oligomers shows that the dependence of the increase in Ca^2+^ influx on GM1 concentration is much higher at a given protein concentration for type A than for type B oligomers (compare Fig. 4C with D). However, this difference appears to result from the different ability of type A oligomers to bind to the membrane with respect to type B oligomers rather than to any difference in the subsequent effects (Fig. 1B).

### Glutamatergic receptors partially mediate the Ca^2+^ influx induced by HypF-N type A oligomers

**Figure 5:**
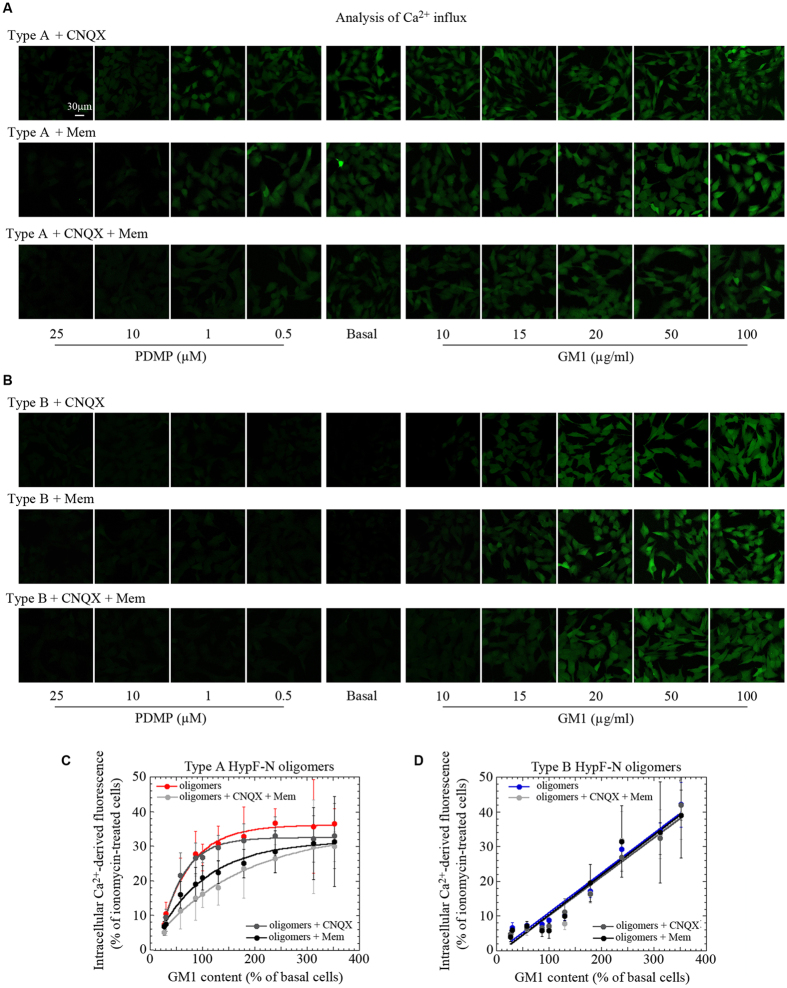
Effects of antagonists on the Ca^2+^ influx induced by HypF-N oligomers. (**A,B**) Representative confocal scanning microscopy images showing levels of intracellular free Ca^2+^ in basal, GM1-enriched (GM1) and GM1-depleted (PDMP) cells treated for 1 h with 12 μM (monomer concentration) type A or type B oligomers in the presence of CNQX (first rows) or mem (second rows). The cells were also treated with type A or type B oligomers in the presence of both CNQX and mem (third rows). (**C,D**) Plots showing the fluorescence associated with intracellular Ca^2+^ *versus* GM1 content after exposure of the cells to type A (**B**, red) or type B oligomers (**C**, blue) in the presence of CNQX (dark gray) or mem (black) or both (pale grey). The corresponding lines represent the best fits to hyperbolic (**B**) and linear (**C**) functions.

The influx of Ca^2+^ caused by protein misfolded oligomers bound to the membrane could be non-specific, involving only the lipid component or it could involve specific membrane receptors. It has been reported that misfolded protein oligomers trigger Ca^2+^ influx by both channel-independent and channel-dependent disruption of the selective permeability of lipid bilayers^28^,^29^,^30^,^34^. To investigate whether or not glutamatergic receptors are involved in the GM1 modulation of the cellular response to the different types of oligomers, the cells were pre-incubated for 10 min with 5.0 μM 6-cyano-7-nitroquinoxaline-2,3-dione (CNQX), a competitive antagonist of α-amino-3-hydroxy-5-methyl-4-isoxazolepropionic acid receptors (AMPA-R), or with 10 μM memantine (mem), a low-affinity antagonist of N-methyl-D-aspartate receptors (NMDA-R). Both CNQX and mem modified slightly, but not significantly, the intracellular Ca^2+^ influx in GM1-modulated cells treated for 1 h with 12 μM type A oligomers, suggesting that there is at least some involvement of both AMPA-R and NMDA-R in the disruption of Ca^2+^ homeostasis by these oligomers (compare Fig. 5A with controls shown in the upper images of Fig. 4A). In addition to the moderate reduction of Ca^2+^ influx into GM1-enriched cells, treatment with mem reduced slightly the Ca^2+^ influx into GM1-depleted cells with respect to the flux recorded in the absence of mem (Fig. 5A,C), again suggesting that NMDA channels are specifically involved in the disruption of Ca^2+^ homeostasis.

We also explored if CNQX and mem displayed addictive effects by incubating cells having different levels of membrane GM1 with a combination of these two antagonists before treatment with the oligomers. The results showed that co-incubation with both antagonists resulted in the summation of their individual effects (Fig. 5A,C), suggesting that the oligomers interfere with both Ca^2+^ channels independently without potentiation. However, even in the presence of both CNQX and mem, the hyperbolic dependence of Ca^2+^ influx on GM1 content was largely maintained, suggesting that a channel-independent mechanism is the primary contribution for the increased cell permeability to Ca^2+^ caused by type A oligomers. By contrast, intracellular Ca^2+^ influx into cells exposed to 12 μM type B oligomers for 1 h was not affected by cell pre-treatment with CNQX or mem or with the two compounds together (Fig. 5B,D), suggesting that there are significant differences in the effects of the two types of oligomers on these receptors.

### A mathematical model of the relationship between Ca^2+^ influx and membrane GM1 levels

Taking into account the data on Ca^2+^ influx and its dependence on GM1 levels, we developed a mathematical model that incorporates all the experimentally observed effects of type A and type B oligomers on these phenomena (Figs 1, 3, 4, 5 and S2–4). In this model, we assume that type A oligomers bind to the membrane of SH-SY5Y cells, and that such binding is mediated by GM1 through the reaction:


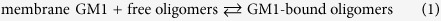


whose dissociation constant (K_D_) is:


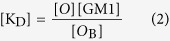


where [O], [GM1] and [O_B_] are the concentrations of free oligomers, of membrane GM1 and of GM1-bound oligomers, respectively. GM1-bound oligomers can be considered to be membrane-bound oligomers through their interaction with GM1. Our data support the view that GM1-bound oligomers are the main species responsible for Ca^2+^ influx and the ensuing disruption of cellular function. Ca^2+^ influx is assumed to occur both via destabilization of the lipid bilayers by bound oligomers, and by effects of this destabilization on the activity of NMDA, AMPA and perhaps other receptors acting as Ca^2+^ channels. In the presence of an excess of GM1, the number of active oligomers becomes the factor limiting the number of oligomer-GM1 interactions as all oligomers are bound to GM1. Under these conditions, any further addition of GM1 does not increase the number of membrane-bound oligomers and consequently does not alter the extent of Ca^2+^ influx.

In more quantitative terms, since the concentration of free oligomers, [O], equals the total concentration of oligomers, [O_T_], minus the concentration of GM1-bound oligomers, [O_B_], equation (2) becomes:


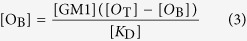


and then:


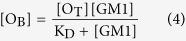


which is an expression similar to the Michaelis-Menten equation for enzyme kinetics. In fact, the rate of Ca^2+^ influx (*v*_Ca_) is assumed to be linearly related to [O_B_], and since the level of cytosolic Ca^2+^ detected in our experiments is far below the equilibrium concentration that can be reached in the case of a highly permeabilized membrane treated with ionomycin, the Ca^2+^-derived fluorescence (F_Ca_) will also be linearly related to *v*_Ca_. As a consequence, F_Ca_ is linearly related to [O_B_]:


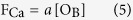


where *a* is the proportionality constant. By combining equations (4) and (5) we obtain:


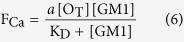


In the case of type B oligomers, the affinity between GM1 and the oligomers appears to be lower, i.e., it displays a much higher K_D_ value and it does not involve receptors. The value of K_D_ + [GM1] in the denominator of equation (6) is, therefore, dominated by the high value of K_D_, and the concentration of GM1 becomes negligible. Thus, equation (6) becomes:


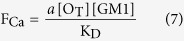


showing that F_Ca_ is linearly related to [GM1] with a slope of *a* [O_T_]/K_D._

Thus, equations (6) and (7) were used to fit all the experimental plots of the fluorescence attributable to intracellular Ca^2+^ (F_Ca_) *versus* GM1 content ([GM1]) reported in this paper for type A and type B oligomers, respectively (Figs 1, 3, 4, 5). The levels of the fits are very high and are able to rationalize all the experimental observations. For type A oligomers the calculated K_D_ value under our experimental conditions was 127 (±12)% [GM1], whereas for type B oligomers the value was more than 200% [GM1]. Since [GM1] is ca. 100 nM in the brain^35^, and 1.25 nM in our experimental conditions after considering the volume fractions occupied by the cells and the extracellular medium, the K_D_ values in tissue can be estimated as 1.59 ± 0.15 nM and >2.5 nM for type A and type B oligomers, respectively.

This model embodied in equations (6) and (7) can explain very closely all our experimental observations involving the effects of type A and type B HypF-N oligomers on the influx of extracellular Ca^2+^ into cells and its dependence on GM1 content (Figs 1, 3 and 5). These observations include the reason why: (i) the dependence of the intracellular Ca^2+^-derived fluorescence (F_Ca_) on GM1 levels for HypF-N type A oligomers is hyperbolic, as equation (6) is describing a hyperbolic function; (ii) F_Ca_ does not increase at high [GM1], as equation (6) becomes F_Ca_ = *a* [O_T_]; (iii) F_Ca_ approaches zero when [GM1] = 0, as in this case equation (6) becomes *F*_Ca_ = 0; (iv) changing the HypF-N concentration from 6.0 μM to 3.0 μM leads to the F_Ca_ value being reduced by a fraction of two at all GM1 concentrations, whereas the hyperbolic behavior does not change, as [O_T_] in equation (6) is reduced two fold; (v) the dependence of F_Ca_ on [GM1] for HypF-N type B oligomers is linear, as equation (7) is the equation of a straight line; and (vi) reducing [HypF-N] by a fraction of two (from 6.0 to 3.0 μM) results in a two fold reduction of the slope in the plot, as [O_T_] in equation (7) is also reduced two fold.

### Experimental measurement of the K_D_ value for the GM1-bound HypF-N oligomers

**Figure 6:**
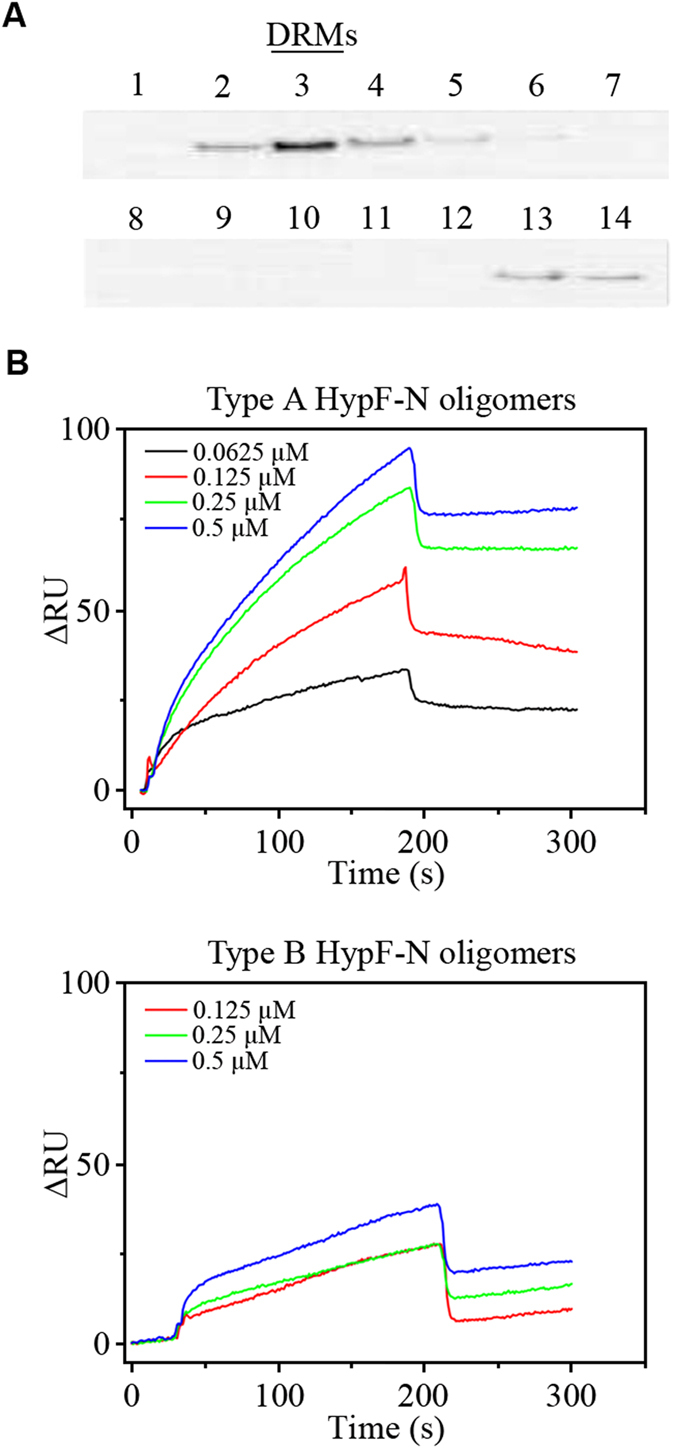
SPR measurements of the affinity of HypF-N type A and type B oligomers for immobilized DRMs. (**A**) Representative immunoblot analysis of flotillin-1 distributions in 14 sucrose gradient fractions collected from the top (low density) to the bottom (high density) of the gradient tube of SH-SY5Y cells with basal GM1 content. A minor flotillin-1 reactivity appears in the two bottom fractions containing the major insoluble components of membranes. (**B**) Sensorgrams for type A (upper traces) or type B (lower traces) oligomers obtained by injecting the two oligomer types at the concentrations indicated into DRMs adsorbed onto SRP chips.

In order to validate experimentally the model described in the previous section, we determined the K_D_ values for the interaction of membrane GM1 with each of the two types of HypF-N oligomers. To this purpose, we used Surface Plasmon Resonance (SPR) to monitor directly the interaction between the detergent resistant fraction of cell membranes (DRMs), containing physiological levels of GM1, with both types of HypF-N oligomers. DRMs were purified from SH-SY5Y cells by ultracentrifugation and the fractions containing DRMs were identified with an immunoblot analysis using anti-flotillin-1 antibodies (Fig. 6A), which recognise flotillin-1, a lipid raft component and caveolae-associated protein. The fraction 3 rich in flotillin-1 was selected as DRMs. The association and dissociation rate constants (k_a_ and k_d_, respectively), as well as the equilibrium association and dissociation constants (K_A_ and K_D_, respectively), were estimated by fitting the SPR titration curves using the Langmuir interaction model (Fig. 6B). Type A and type B oligomers showed similar k_a_ values (1.4(±0.3)10^5^ and 3.4(±0.5)10^5 ^M^−1^s^−1^), indicating a modest association rate to the immobilized DRMs. A difference of two orders of magnitude in k_d_ values was, however, found (2.0(±0.2)10^−4 ^s^−1^ and 1.2(±0.1)10^−2 ^s^−1^, respectively), confirming the higher affinity of type A oligomers with respect to type B oligomers for the immobilized DRMs (K_D_ values of 1.5(±0.2)10^−9 ^M and 4.4(±0.5)10^−8 ^M, respectively). Such experimentally determined values of K_D_ are in good agreement with those obtained from the model (1.59(±0.15) 10^−9 ^M and >2.5 10^−9 ^M for type A and B oligomers, respectively) thus validating the model itself.

## Discussion

Our results show that the two pairs of toxic and nontoxic oligomers obtained from different protein systems, namely HypF-N and Aβ_42_, behave with close similarity under the same conditions. In particular, we found similar quantitative relationships between the GM1 content of the cell membrane and the ability of the membrane to bind oligomers from different peptides/proteins but with the same level of toxicity. However, the relationship appears different for type A and type B HypF-N oligomers, as well as for A+ and A− Aβ_42_ oligomers, that is for oligomers of the same peptide/protein but exhibiting different levels of toxicity. In fact, it appears that HypF-N type A (or Aβ_42_ A+) oligomer binding to the cell membrane occurs with high affinity and saturation at high GM1 levels, whereas the GM1-dependence of HypF-N type B (or Aβ_42_ A−) oligomer binding shows lower affinity and is apparently linear. Very similar trends have been observed for all measures of the cell toxicity that result from oligomer binding, including Ca^2+^ influx, calcein release and MTT reduction, indicating that the observed cytotoxicity is closely related to the affinity to the membrane of a given type of oligomer.

We have derived a method for the quantitative analysis of these findings, as described in the *Results* section and embodied in equations (6) and (7), that is able to describe in detail all the experimental observations reported in this paper. These include the effects of the HypF-N type A and type B oligomers on the influx of the extracellular Ca^2+^ inside the exposed cells and its dependence on GM1 content, protein concentration and the perturbations resulting from the treatment with specific inhibitors of AMPA-R and NMDA-R. This analysis allows the K_D_ value of the binding of the oligomers to GM1 within the membrane to be determined with a high degree of accuracy. In the case of the HypF-N type A oligomers, we have shown that the K_D_ value determined in this way is very close to that measured experimentally using SPR and immobilized DRMs taken from cells.

Overall, this analysis has provided a robust basis for the interpretation of the evidence obtained in this study of the important role performed by membrane GM1 as a key site for binding the oligomeric species that results in cellular impairment through perturbations of Ca^2+^ homeostasis and membrane-bound receptors. However, no common consensus so far has been reached on the molecular details of membrane disruption^36^. The influx of Ca^2+^ caused by protein misfolded oligomers bound to the membrane could be non-specific, involving just lipid component or specific membrane receptors^28^,^29^,^30^,^34^. A number of experimental findings indicate that amyloid aggregates, particularly oligomers, can also interact selectively with voltage-dependent Ca^2+^ channels or glutamatergic receptors^37^,^38^. These receptors are required for synaptic targeting of toxic Aβ oligomers^19^, which results in a rise in intracellular Ca^2+^ followed by other chemical changes, eventually leading to dendritic dystrophy and neurodegeneration^39^. It has therefore been suggested that Aβ oligomers bind to synapses in close proximity to NMDA and AMPA receptors, whose deregulation disrupts Ca^2+^ homeostasis, providing a molecular basis for plasticity failure, synapse loss and memory dysfunction in AD^40^. However, other cell surface proteins have been considered as possible candidate receptors of Aβ oligomers, including APP^41^, TNFR1 (tumor-necrosis factor receptor-1)^42^, the receptor for advanced glycation end products (RAGE)^43^, the non-infectious form of the prion protein (PrPc)^44^.

Our findings are of interest to re-evaluate the conclusions from studies of the effects of oligomers with PrP^c^^45^. There is strong evidence of a key role for PrP^c^, a protein that is associated with lipid rafts, as a receptor for oligomers of the Aβ peptide, resulting in the activation of a Fyn-mediated complex signaling cascade leading to tau phosphorylation and loss of Ca^2+^ homeostasis^45^. More recently, it has been reported that the extent of the interaction of Aβ oligomers with PrP^c^ is at least as accurate as any other predictor of memory impairment in AD mouse models and human AD patients. This study also reported that the fraction of total Aβ oligomers interacting with PrP^c^ varied considerably in different AD models and could determine the extent of the contribution to AD-like symptoms of PrP^c^-dependent molecular mechanisms^46^. The data we reported here, along with conclusions from several other studies^20^,^21^,^47^, support the idea that the oligomers we have used can interact with membranes in other ways, notably through direct binding to GM1. This then results in the disruption of lipid bilayers, alteration of their permeability and the misfunction of raft-associated Ca^2+^ channels, leading to Ca^2+^ influx into cells. Although several studies have shown the lack of any role of PrPc-Aβ oligomer interaction as a determinant of the AD phenotype^48^,^49^, our findings do not necessarily contradict the view that PrP^c^ behaves as a receptor of a class of Aβ oligomers. In fact, it could well be that due to the close vicinity between PrP^c^ and GM1, oligomer interaction with the latter could be needed to induce structural perturbations in PrP^c^ resulting in the described effects of the Fyn-mediated signaling cascade. Alternatively, as recently reported^46^, considering the ambiguous and generic significance of the term “oligomers”, including many structurally distinct aggregated entities, different protein oligomers, including our Aβ aggregates, could interact with PrP^c^ and GM1 with different and inversely related affinities; in our case, the oligomers we used could be able to interact with GM1 but not with PrP^c^. This consideration is supported by the same authors when they conclude that other forms of Aβ oligomers with lower affinity to PrP^c^ may also contribute to AD progression. Overall, the question of the physiological interactor(s) of Aβ oligomers in brain tissue needs further investigation.

The findings of the present study also provide important information relating to the vulnerabilities of different cell types to the same oligomers^21^,^50^ and of the same cell types in different functional states^51^. It is increasingly evident that toxicity is not a feature that is inherent to a given type of misfolded protein oligomer, but is instead a property that emerges from the complex interplay between the structural and physicochemical features of oligomers and of the specific characteristics of the membranes with which they interact^6^. Further studies are needed to assess in greater detail the importance of our data in a more physiological context.

Finally, and perhaps even more importantly, the findings reported in this study indicate that the susceptibility of cells to the effects of misfolded oligomeric assemblies is directly related to the oligomer binding affinity to the cell membrane. Previous studies have shown that this affinity is dependent on the size of the oligomers and the extent of solvent-exposed hydrophobicity^10^,^12^,^17^,^52^. The present results demonstrate, however, that once they are bound, different types of oligomers generate similar toxic effects, indicating that the cytotoxicity of misfolded proteins is not simply a characteristic of a given polypeptide sequence but a result of a complex interplay between the physicochemical features of both oligomers and membranes.

## Materials and Methods

### Modulation and evaluation of the GM1 content of neuronal cells

Human neuroblastoma SH-SY5Y cells (A.T.C.C. Manassas, VA) were cultured in Dulbecco’s Modified Eagle’s Medium (DMEM), F-12 Ham with 25 mM HEPES and NaHCO_3_ (1:1) supplemented with 10% fetal bovine serum (FBS), 1.0 mM glutamine and 1.0% penicillin and streptomycin solution. Cells were maintained in a 5.0% CO_2_ humidified atmosphere at 37 °C and grown until 80% confluence for a maximum of 20 passages. The SH-SY5Y cell culture media were supplemented with 0.5, 1.0, 10 or 25 μM PDMP, or with 10, 15, 20, 50 or 100 μg/ml GM1 from bovine brain (Sigma Aldrich, Saint Louis, MO, USA). After incubation for 48 h at 37 °C the cells were loaded with 2.25 μg/ml CTX-B and analysed using a FACSCanto flow cytometer (BD Biosciences, San Jose, CA). The measured fluorescence intensities were expressed as fractional changes above the resting baseline, ΔF/F^20^. The GM1 content of the membrane was also measured in SH-SY5Y cells seeded on glass coverslips treated with 4.5 μg/ml Alexa Fluor 647-conjugated CTX-B or, alternatively, with 1:100 diluted rabbit polyclonal anti-GM1 antibodies (Calbiochem, EMD Chemicals Inc., Darmstadt, Germany) and then 1:1000 diluted Alexa Fluor 488-conjugated anti-rabbit secondary antibodies (Life Technologies, CA, USA). The emitted fluorescence was detected after excitation at 647 and 488 nm, respectively, by a TCS SP5 scanning confocal microscopy system (Leica, Mannheim, Germany) equipped with an argon laser source. A series of 1.0 μm thick optical sections (1024 × 1024 pixels) was taken through the cell depth for each sample using a Leica Plan Apo 63× oil immersion objective and projected as a single composite image by superimposition (Leica, Mannheim, Germany).

### Preparation of HypF-N and Aβ_42_ oligomers

HypF-N and Aβ_42_ oligomers were prepared as described previously^10^,^12^. In brief, the HypF-N protein stock solution was diluted to a concentration of 48 μM in 50 mM acetate buffer, 12% (v/v) trifluoroethanol (TFE), 2.0 mM dithiothreitol (DTT), pH 5.5 (condition A), or in 20 mM trifluoroacetic acid (TFA), 330 mM NaCl, pH 1.7 (condition B). The resulting samples were incubated for 4 h at 25 °C and then centrifuged at 16,100 r.c.f.; the pellet was dried under N_2_ to remove residual TFE or TFA, dissolved in DMEM at a monomer equivalent concentration of 12 μM and then added to SH-SY5Y cells to give a series of different final protein concentrations. No significant destabilisation of the oligomers or change in their structures or morphologies could be detected after this procedure, as previously reported^10^. The lyophilised Aβ_42_ peptide (Sigma Aldrich, Saint Louis, MO, USA) was dissolved in 100% hexafluoro-2-isopropanol (HFIP) to 1.0 mM and then the solvent was evaporated. Aβ_42_ oligomers were prepared by suspending the peptide at the same concentration in 50 mM NaOH and diluting this solution in PBS to give a final Aβ_42_ concentration of 25 μM. Then, the sample was centrifuged at 22,000 r.c.f. for 30 min, the pellet discarded and the supernatant incubated at 25 °C without agitation for 1 day to obtain A+ oligomers or for 4 days to obtain A− oligomers^12^.

### Analysis of oligomer binding to cell membranes

SH-SY5Y cells, with a given GM1 membrane content, were seeded on glass coverslips and treated for 1 h with 12 μM (monomer equivalent) type A or type B HypF-N oligomers^10^ or with 10 μM (monomer equivalent) A+ or A− Aβ_42_ oligomers^12^. After incubation, the cells were counterstained with 5.0 μg/ml Alexa Fluor 633-conjugated wheat germ agglutinin. The presence of oligomers was detected with 1:1000 diluted rabbit polyclonal anti-HypF-N antibodies (Primm, Milan, Italy) or with 1:800 diluted mouse monoclonal anti-Aβ_42_ antibodies (Signet, Dedham, MA, USA), as appropriate, and subsequently with 1:1000 diluted Alexa Fluor 488-conjugated anti-mouse secondary antibodies (Life Technologies, CA, USA). To detect only the oligomers bound to the cell surface, the cellular membrane was not permeabilised at this stage, thus preventing antibody internalisation. Fluorescence emission was detected after double excitation at 488 nm and 633 nm by a TCS SP5 scanning confocal microscopy system. A series of 1.0 μm thick optical sections (1024 × 1024 pixels) was taken through the cell depth for each sample using a Leica Plan Apo 63× oil immersion objective and all sections were projected as a single composite image by superimposition. The co-localisation of oligomers (small dots) and cell membrane was estimated for regions of interest in 12–22 cells, in three different experiments, using ImageJ and JACOP plugin (http://www.rsb.info.nih.gov) software and expressed as fraction of oligomer binding by the PCC relative to cells treated with 100 μg/ml GM1. A very few proportion of aggregates resulting from clustering of oligomers and appearing as large dots on the cellular membranes were excluded from the quantitative analysis of florescence signals.

In order to investigate whether endocytotic processes, sorting and degradation occur in our experimental conditions, cells with basal and increased GM1 content (100 μg/ml GM1) were treated with A+ Aβ_42_ oligomers (10 μM, monomer equivalents) for 15 min at 37 °C, 60 min at 37 °C or 60 min at 16 °C. In addition, to evaluate the presence of intracellular fluorescence, in a set of experiments, three median planes of cells with increased GM1 content (100 μg/ml GM1) treated for 1 h with A+ or A− Aβ_42_ oligomers (10 μM, monomer equivalents) were projected as a single composite image by superimposition.

### Measurements of cytosolic Ca^2+^ levels

The cytosolic Ca^2+^ levels in the cells, loaded with 4.0 μM Fluo3-AM, were measured by scanning confocal fluorescence microscopy after excitation at 488 nm^20^. 10–22 cells, in three different experiments, were analysed using ImageJ software. The fluorescence intensities were expressed as the percentage of that measured in 1.0 μM ionomycin-treated cells^53^. SH-SY5Y cells, whose GM1 content had been modulated as described above, were cultured on glass coverslips and treated for 1 h with 3.0, 6.0 or 12 μM type A or type B HypF-N oligomers (monomer equivalents). In a separate set of experiments, the cells were treated for 10 min with 5.0 μM CNQX^54^, with 10 μM mem^55^, or with both reagents, prior to exposure to oligomers.

### Measurements of membrane permeability

SH-SY5Y cells, whose GM1 content had been modulated as described above, were cultured on glass coverslips, loaded with 1.0 μM calcein-AM for 10 min at 37 °C and then treated separately with each type of HypF-N oligomers (12 μM, monomer equivalent) for 1 h. The emitted fluorescence was detected after excitation at 488 nm by the confocal scanning system. To quantify the fluorescence intensity of calcein, variable numbers of cells (10 to 22) were analysed in each experiment using the ImageJ software (NIH). The fluorescence intensities were expressed as fractional changes above the resting baseline for untreated cells, ΔF/F^20^,^33^.

### MTT reduction test

Aggregate cytotoxicity was assessed in SH-SY5Y cells seeded in 96-well plates by means of the 3-(4,5-dimethylthiazol-2-yl)-2,5-diphenyltetrazolium bromide (MTT) assay^10^. The cells, with GM1 content modulated as described above, were treated for 24 h with either HypF-N oligomers (12 μM, monomer equivalents), and then incubated with 0.5 mg/ml MTT at 37 °C for 4 h, and with cell lysis buffer (20% SDS, 50% N,N-dimethylformamide, pH 4.7) for 3 h. The absorbance values of blue formazan were determined at 590 nm. Cell viability was expressed as the percentage of MTT reduction in treated cells as compared to untreated cells.

### Surface plasmon resonance

The SH-SY5Y cells were washed twice with ice-cold PBS containing 0.4 mM Na_3_VO_4_, scraped, and collected by centrifugation at 1000xg for 10 min. In order to purify DRMs, the cells were dispersed in 10 mM Tris–HCl buffer, pH 7.5, containing 150 mM NaCl, 5.0 mM EDTA, 1.0 mM Na_3_VO_4_, 1.0% Triton X-100 (TNE buffer) with protease inhibitors, disrupted in a Dounce homogenizer and centrifuged at 1,500 × g for 5 min at 4.0 °C, as previously reported^31^. Briefly, the post-nuclear lysate was adjusted to contain 40% (w/v) sucrose by 1:1 addition of 80% sucrose prepared in TNE buffer, placed at the bottom of an ultracentrifuge tube and overlaid with two layers of 30% and 5.0% sucrose in TNE buffer. The sucrose gradient was centrifuged at 170,000 × g for 18 h at 4.0 °C using a Beckman SW40Ti rotor (Beckman Coulter, CA, USA). Then the fractions were collected from the top of the gradient as follows: 1.0 ml for fraction 1, 0.50 ml for fractions 2 to 11, and 2.0 ml for fractions 12 and 13, while the pellet was dissolved in 0.08 ml of TNE buffer (fraction 14). A representative amount of each fraction was subjected to immunoblot analysis of flotillin-1 on a 12% (w/v) SDS/PAGE, blotted onto a PVDF membrane, incubated with 1:500 mouse monoclonal primary antibody (Beckton Dickinson Bioscences, San Diego, CA) and an anti-mouse secondary antibody. Fraction 3, rich in flotillin-1 (DRMs), was collected and extensively dialysed against TNE buffer to remove sucrose.

SPR measurements were carried out by means of a Biacore X™ using bare gold sensor chips (General Electric Healthcare Bio-Sciences AB, Uppsala, Sweden). The DRMs were incubated on the surface of the chips overnight in a wet chamber. Then the modified sensor chips were washed with 20 mM phosphate buffer, pH 7.0 and placed into the instrument. The reaction was evaluated by injecting type A or type B HypF-N oligomers at different concentrations, from 0 to 0.5 μM, prepared in 20 mM phosphate, pH 7.0 (the association phase). The same solution without oligomers was also used as running buffer (dissociation phase). All the buffers were filtered (0.22 μm) prior to use. All measurements were performed at 25 °C, using the following experimental parameters: association phase 180 s, dissociation phase 180 s, flow 5.0 μL/min. The resulting sensorgrams were analysed by means of the BIAevalutation 3.1 software.

### Statistical analysis

All data were expressed as means ± standard deviations (SD). Comparisons between the different groups were performed by ANOVA followed by Bonferroni’s post comparison test.

## Additional Information

**How to cite this article**: Evangelisti, E. *et al*. Binding affinity of amyloid oligomers to cellular membranes is a generic indicator of cellular dysfunction in protein misfolding diseases. *Sci. Rep.* **6**, 32721; doi: 10.1038/srep32721 (2016).

## Supplementary Material

Supplementary Information

## References

[b1] Bemporad F. & Chiti F. Protein misfolded oligomers: experimental approaches, mechanism of formation, and structure-toxicity relationships. Chem. Biol. 19, 315–327 (2012). 22444587 10.1016/j.chembiol.2012.02.003

[b2] Martins I. C. . Lipids revert inert Abeta amyloid fibrils to neurotoxic protofibrils that affect learning in mice. EMBO J. 27, 224–233 (2008). 18059472 10.1038/sj.emboj.7601953 PMC2206134

[b3] Cohen S. I. .Proliferation of amyloid-β42 aggregates occurs through a secondary nucleation mechanism. Proc. Natl. Acad. Sci. USA. 110, 9758–9763 (2013). 23703910 10.1073/pnas.1218402110 PMC3683769

[b4] Kinnunen P. J. K. Amyloid formation on lipid membrane surfaces. Open Biol. J. 2, 163–175 (2009).

[b5] Lukiw W. J. Alzheimer’s disease (AD) as a disorder of the plasma membrane. Front Physiol. 4, 24 (2013). 23424582 10.3389/fphys.2013.00024 PMC3573332

[b6] Cecchi C. & Stefani M. The amyloid-cell membrane system. The interplay between the biophysical features of oligomers/fibrils and cell membrane defines amyloid toxicity. Biophys. Chem. 182, 30–43 (2013). 23820236 10.1016/j.bpc.2013.06.003

[b7] Bucciantini M. . Inherent toxicity of aggregates implies a common mechanism for protein misfolding diseases. Nature 416, 507–511 (2002). 11932737 10.1038/416507a

[b8] Glabe C. G. & Kayed R. Common structure and toxic function of amyloid oligomers implies a common mechanism of pathogenesis. Neurology 66, S74–S78 (2006). 16432151 10.1212/01.wnl.0000192103.24796.42

[b9] Knowles T. P., Vendruscolo M. & Dobson C. M. The amyloid state and its association with protein misfolding diseases. Nat. Rev. Mol. Cell. Biol. 15, 384–396 (2014). 24854788 10.1038/nrm3810

[b10] Campioni S. . A causative link between the structure of aberrant protein oligomers and their toxicity. Nat. Chem. Biol. 6, 140–147 (2010). 20081829 10.1038/nchembio.283

[b11] Cremades N. . Direct observation of the interconversion of normal and toxic forms of α–synuclein. Cell 149, 1048–1059 (2012). 22632969 10.1016/j.cell.2012.03.037 PMC3383996

[b12] Ladiwala A. R. . Conformational differences between two amyloid beta oligomers of similar size and dissimilar toxicity. J. Biol. Chem. 287, 24765–24773 (2012). 22547072 10.1074/jbc.M111.329763 PMC3397903

[b13] Krishnan R. . Conserved features of intermediates in amyloid assembly determine their benign or toxic states. Proc. Natl. Acad. Sci. USA. 109, 11172–11177 (2012). 22745165 10.1073/pnas.1209527109 PMC3396487

[b14] Liu P. . Quaternary structure defines a large class of amyloid-β oligomers neutralized by sequestration. Cell Rep. 11, 1760–1771 (2015). 26051935 10.1016/j.celrep.2015.05.021 PMC4494129

[b15] Zampagni M. . A comparison of the biochemical modifications caused by toxic and non-toxic protein oligomers in cells. J. Cell. Mol. Med. 15, 2106–2116 (2011). 21155974 10.1111/j.1582-4934.2010.01239.x PMC4394221

[b16] Tatini F. . Amyloid-β oligomer synaptotoxicity is mimicked by oligomers of the model protein HypF-N. Neurobiol. Aging 34, 2100–2109 (2013). 23601807 10.1016/j.neurobiolaging.2013.03.020

[b17] Mannini B. . Toxicity of protein oligomers is rationalized by a function combining size and surface hydrophobicity. ACS Chem. Biol. 9, 2309–2317 (2014). 25079908 10.1021/cb500505m

[b18] Diaz J. C., Simakova O., Jacobson K. A. Arispe & Pollard H. B. N. Small molecule blockers of the Alzheimer Abeta calcium channel potently protect neurons from Abeta cytotoxicity. Proc. Natl. Acad. Sci. USA 106, 3348–3353 (2009). 19204293 10.1073/pnas.0813355106 PMC2637905

[b19] Decker H. . N-methyl-D-aspartate receptors are required for synaptic targeting of Alzheimer’s toxic amyloid-beta peptide oligomers. J. Neurochem. 115, 1520–1529 (2010). 20950339 10.1111/j.1471-4159.2010.07058.x

[b20] Evangelisti E. . Membrane lipid composition and its physicochemical properties define cell vulnerability to aberrant protein oligomers. J. Cell. Sci. 125, 2416–2427 (2012). 22344258 10.1242/jcs.098434

[b21] Malchiodi-Albedi F. . Lipid raft disruption protects mature neurons against amyloid oligomer toxicity. Biochim. Biophys. Acta 1802, 406–415 (2010). 20060899 10.1016/j.bbadis.2010.01.007

[b22] Schengrund C. L. Lipid rafts: keys to neurodegeneration. Brain Res. Bull. 82, 7–17 (2010). 20206240 10.1016/j.brainresbull.2010.02.013

[b23] Pernber Z., Blennow K., Bogdanovic N., Mansson J. E. & Blomqvist M. Altered distribution of the gangliosides GM1 and GM2 in Alzheimer’s disease. Dement Geriatr. Cogn. Disord. 33, 174–188 (2012). 22572791 10.1159/000338181

[b24] Yamamoto N., Matsubara T., Sato T. & Yanagisawa K. Age-dependent high-density clustering of GM1 ganglioside at presynaptic neuritic terminals promotes amyloid beta-protein fibrillogenesis. Biochim. Biophys. Acta 1778, 2717–2726 (2008). 18727916 10.1016/j.bbamem.2008.07.028

[b25] Wakabayashi M. & Matsuzaki K. Ganglioside-induced amyloid formation by human islet amyloid polypeptide in lipid rafts. FEBS Lett. 583, 2854–2858 (2009). 19647738 10.1016/j.febslet.2009.07.044

[b26] McLean C. A. . Soluble pool of Abeta amyloid as a determinant of severity of neurodegeneration in Alzheimer’s disease. Ann Neurol. 46, 860–866 (1999). 10589538 10.1002/1531-8249(199912)46:6<860::aid-ana8>3.0.co;2-m

[b27] Tamboli I. Y., Prager K., Barth E., Heneka M., Sandhoff K. & Walter J. Inhibition of glycosphingolipid biosynthesis reduces secretion of the beta-amyloid precursor protein and amyloid beta-peptide. J. Biol. Chem. 280, 28110–28117 (2005). 15923191 10.1074/jbc.M414525200

[b28] Marx J. Alzheimer’s disease. Fresh evidence points to an old suspect: calcium. Science 318, 384–385 (2007). 17947560 10.1126/science.318.5849.384

[b29] Bojarski L., Herms J. & Kuznicki J. Calcium dysregulation in Alzheimer’s disease. Neurochem Int. 52, 621–633 (2008). 18035450 10.1016/j.neuint.2007.10.002

[b30] Demuro A., Parker I. & Stutzmann G. E. Calcium signaling and amyloid toxicity in Alzheimer disease. J. Biol. Chem. 285, 12463–12468 (2010). 20212036 10.1074/jbc.R109.080895 PMC2857063

[b31] Orrenius S., Zhivotovsky B. & Nicotera P. Regulation of cell death: the calcium-apoptosis link. Nat. Rev. Mol. Cell. Bio. 4, 552–565 (2003). 12838338 10.1038/nrm1150

[b32] Evangelisti E. . Lipid rafts mediate amyloid-induced calcium dyshomeostasis and oxidative stress in Alzheimer’s disease. Curr. Alzheimer Res. 10, 143–153 (2013). 22950913 10.2174/1567205011310020004

[b33] Papadopoulos N. G., Dedoussis G. V., Spanakos G., Gritzapis A. D., Baxevanis C. N. & Papamichail M. An improved fluorescence assay for the determination of lymphocyte-mediated cytotoxicity using flow cytometry. J. Immunol Meth. 177, 101–111 (1994). 10.1016/0022-1759(94)90147-3 7822816

[b34] Arispe N., Diaz J. C. & Simakova O. Prospects for treating Alzheimer’s disease with Abeta channel blockers. Biochim. Biophys. Acta 1768, 1952–1965 (2007). 17490607 10.1016/j.bbamem.2007.03.014

[b35] Davidsson P., Fredman P. & Svennerholm L. Gangliosides and sulphatide in human cerebrospinal fluid: quantitation with immunoaffinity techniques. J. Chromatogr. 496, 279–289 (1989). 2613833 10.1016/s0378-4347(00)82577-3

[b36] Butterfield S. M. & Lashuel H. A. Amyloidogenic protein–membrane interactions:mechanistic insight from model systems Angew. Chem. Int. Ed. 49, 5628–5654 (2010). 10.1002/anie.200906670 20623810

[b37] Kelly B. I. & Ferreira A. Beta-amyloid-induced dynamin 1 degradation is mediated by N-methyl-D-aspartate receptors in hippocampal neurons. J. Biol. Chem. 281, 28079–28089 (2006). 16864575 10.1074/jbc.M605081200

[b38] Hsieh H. . AMPARremoval underlies Abeta-induced synaptic depression and dendritic spine loss. Neuron 52, 831–843 (2006). 17145504 10.1016/j.neuron.2006.10.035 PMC1850952

[b39] Resende R. . Susceptibility of hippocampal neurons to Abeta peptide toxicity is associated with perturbation of Ca^2+^ homeostasis. Brain Res. 1143, 11–21 (2007). 17336275 10.1016/j.brainres.2007.01.071

[b40] Liu S. J. & Zukin R. S. Ca^2+^ -permeable AMPA receptors in synaptic plasticity and neuronal death, Trends Neurosci. 30, 126–134 (2007). 17275103 10.1016/j.tins.2007.01.006

[b41] Shadek G. M. . Aβ induces cell death by direct interaction with its cognate extracellular domain on APP (APP 597–624). FASEB J. 20, 1254–1266 (2006). 16636103 10.1096/fj.05-5032fje PMC1847355

[b42] He P. . Tumor necrosis factor death receptor signaling cascade is required for amyloid-β protein-induced neuron death. J. Neurosci. 24, 1760–1771 (2004). 14973251 10.1523/JNEUROSCI.4580-03.2004 PMC6730458

[b43] Yan S. D. . RAGE and amyloid-beta peptide neurotoxicity in Alzheimer’s disease. Nature 382, 685–691 (1996). 8751438 10.1038/382685a0

[b44] Lauren J., Gimbel D. A., Nygaard H. B., Gilbert J. W. & Strittmatter S. M. Cellular prion protein mediates impairment of synaptic plasticity by amyloid-β oligomers, Nature 457, 1128–1132 (2009). 19242475 10.1038/nature07761 PMC2748841

[b45] Gunther E. C. & Strittmatter S. M. Alzheimer amyloid-beta oligomer bound to postsynaptic prion protein activates Fyn to impair neurons. Nat. neurosci. 15, 1227–1235 (2012). 22820466 10.1038/nn.3178 PMC3431439

[b46] Kostylev M. A. . Prion-protein-interacting amyloid-β oligomers of high molecular weight are tightly correlated with memory impairment in multiple Alzheimer mouse models. J. Biol. Chem. 290, 17415–17438, (2015). 26018073 10.1074/jbc.M115.643577 PMC4498078

[b47] Hong S. . Soluble Abeta oligomers are rapidly sequestered from brain ASF *in vivo* and bind GM1 ganglioside on cellular membranes. Neuron 82, 308–319 (2014). 24685176 10.1016/j.neuron.2014.02.027 PMC4129520

[b48] Balducci C. . Synthetic amyloid-beta oligomers impair long-term memory independently of cellular prion protein. Proc Natl Acad Sci. 107, 2295–2300 (2010). 20133875 10.1073/pnas.0911829107 PMC2836680

[b49] Cissé M., Sanchez P. E., Kim D. H., Ho K., Yu G. Q. & Mucke L. Ablation of cellular prion protein does not ameliorate abnormal neural network activity or cognitive dysfunction in the J20 line of human amyloid precursor protein transgenic mice. J. Neurosci. 31, 10427–10431 (2011). 21775587 10.1523/JNEUROSCI.1459-11.2011 PMC3314063

[b50] Cecchi C. . Insights into the molecular basis of the differing susceptibility of varying cell types to the toxicity of amyloid aggregates. J. Cell. Sci. 118, 3459–3470 (2005). 16079288 10.1242/jcs.02473

[b51] Cecchi C. . Replicating neuroblastoma cells in different cell cycle phases display different vulnerability to amyloid toxicity. J. Mol. Med. 86, 197–209 (2008). 17885746 10.1007/s00109-007-0265-3

[b52] Cizas P. . Size-dependent neurotoxicity of β-amyloid oligomers. Arch. Biochem. Biophys. 496, 84–92 (2010). 20153288 10.1016/j.abb.2010.02.001 PMC2853175

[b53] Tanimura A., Nezu A., Morita T., Turner R. J. & Tojyo Y. Fluorescent biosensor for quantitative real-time measurements of inositol 1,4,5,-triphosphate in single living cells. J. Biol. Chem. 279, 38095–38098 (2004). 15272011 10.1074/jbc.C400312200

[b54] Younkin D. P. . Inducible expression of neuronal glutamate receptor channels in the NT2 human cell line. Proc. Natl. Acad. Sci. USA 90, 2174–2178 (1993). 7681588 10.1073/pnas.90.6.2174 PMC46048

[b55] De Felice F. G. . Abeta oligomers induce neuronal oxidative stress through an N-methyl-D-aspartate receptor-dependent mechanism that is blocked by the Alzheimer drug memantine. J. Biol. Chem. 282, 11590–11601 (2007). 17308309 10.1074/jbc.M607483200

