# Supplementary material for: Binding affinity of amyloid oligomers to cellular membranes is a generic indicator of cellular dysfunction in protein misfolding diseases
Source: Sci Rep. 2016 Sep 13;6:32721. doi: 10.1038/srep32721 (PMC5020652; doi:10.1038/srep32721)
Supplement: Supplementary Information [file srep32721-s1.pdf]

Binding affinity of amyloid oligomers to cellular membranes is a generic indicator of cellular dysfunction in protein misfolding diseases

Elisa Evangelisti°[1], Roberta Cascella°[1], Matteo Becatti[1], Giovanna Marrazza[2], Christopher M. Dobson[3], Fabrizio Chiti[1], Massimo Stefani[1] and Cristina Cecchi[1,§]

Supplementary Information

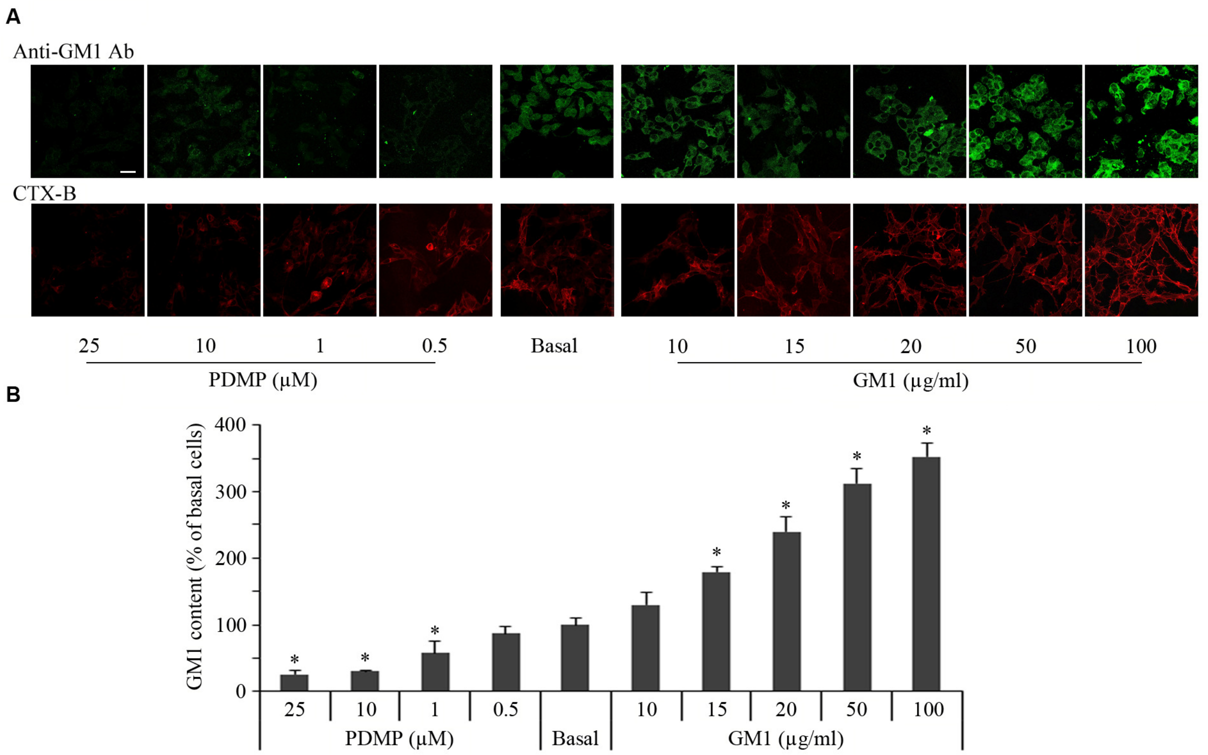

Figure S1. Modulation of GM1 levels in human SH-SY5Y neuroblastoma cells. (A) Representative confocal microscope images of GM1 distribution at the cell surface probed by anti-GM1 antibodies (green) or the CTX-B conjugate (red) in basal cells or in cells pre-treated with the indicated concentrations of PDMP or GM1 for 48 h. (B) Flow cytometric analysis of membrane GM1 content in basal cells or in cells pre-treated with the indicated concentrations of PDMP or GM1 for 48 h. After GM1 modulation, single-cell suspensions were incubated with the fluorescent probe CTX-B. The values reported are means ± SD of four independent experiments and the asterisks indicate a significant difference relative to cells with basal GM1 levels ($p \leq 0.05$).

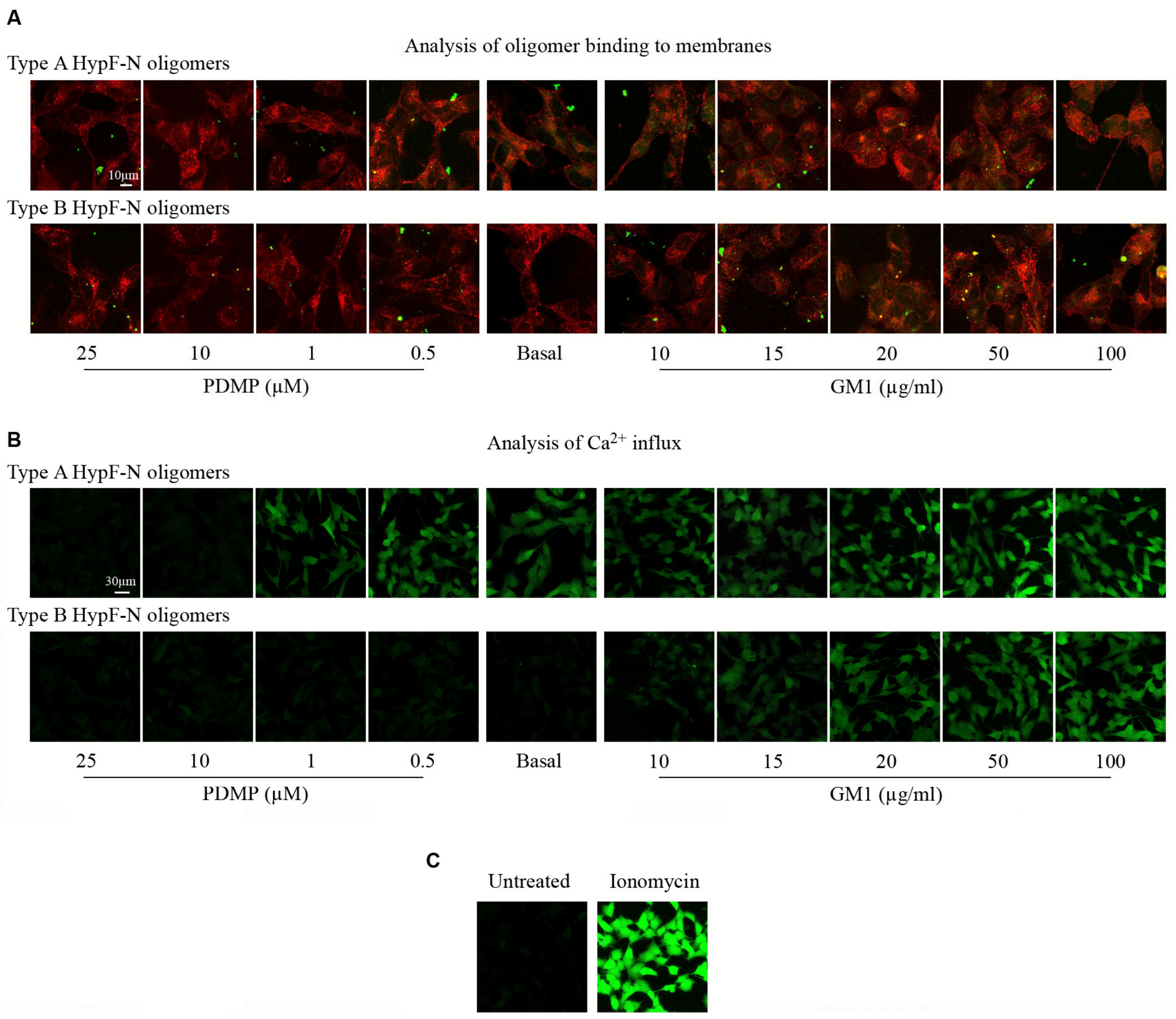

Figure S2. Analysis of the different recruitment of HypF-N oligomers to the plasma membrane and the ensuing Ca^{2+} influx in cells with different GM1 content. (A) Representative confocal scanning microscopy images of GM1-depleted (PDMP), basal and GM1-enriched (GM1) cells treated for 1 h with type A (upper images) or type B (lower images) HypF-N oligomers at 12 μM concentration (monomer equivalent). In all images, red and green fluorescence indicates cell membranes and HypF-N oligomers, respectively. (B) Representative confocal scanning microscopy images of GM1-depleted (PDMP), basal and GM1-enriched (GM1) cells showing levels of intracellular free Ca^{2+} following treatment for 1 h with type A (upper images) or type B (lower images) HypF-N oligomers at 12 μM concentration (monomer equivalent). In all images, the green fluorescence arises from Ca^{2+} binding to the intracellular Fluo3-AM probe. (C) Representative confocal scanning microscopy images of basal cells that were not treated or treated for 1 h with 1.0 μM ionomycin.

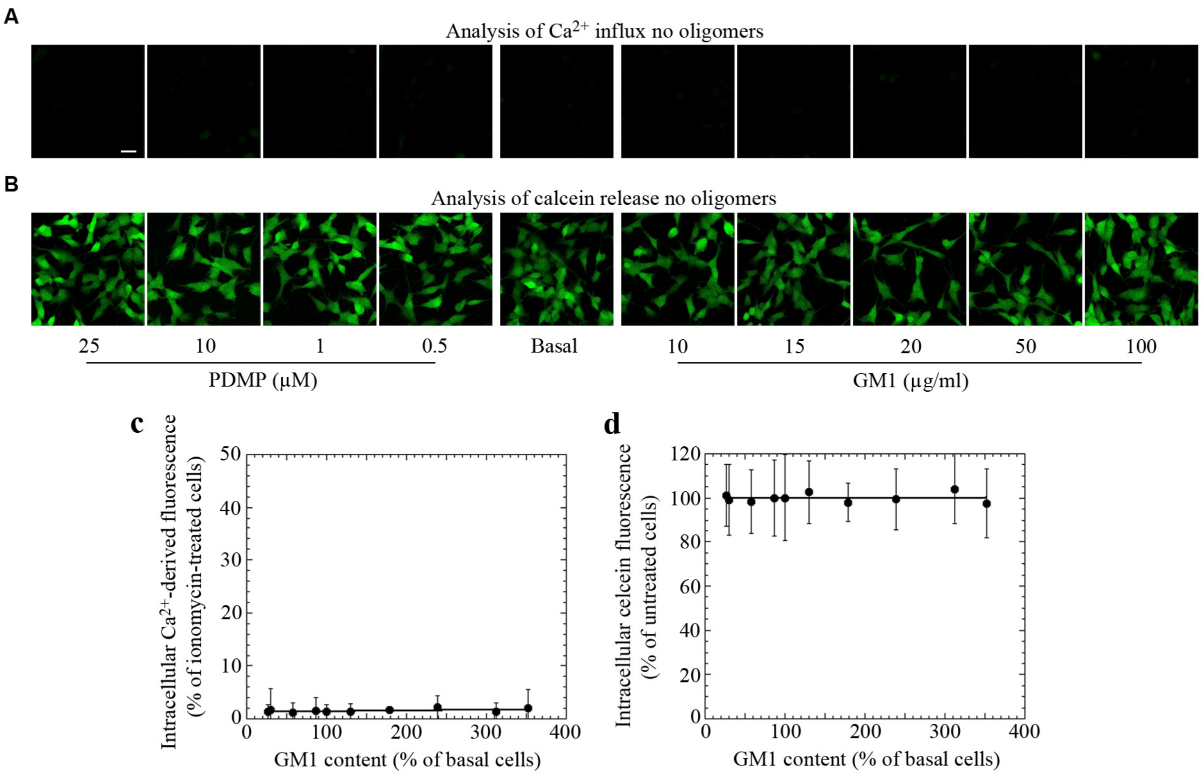

Figure S3. Ca^{2+} influx and membrane permeability in cells with different GM1 contents in the absence of oligomers. (A,B) Confocal scanning microscopy images showing levels of intracellular calcein (A) and free Ca^{2+} (B) in basal, GM1-enriched (GM1) and GM1-depleted (PDMP) cells, incubated in the absence of oligomers. (C,D) Plots showing the fluorescence associated with intracellular Ca^{2+} (C) or intracellular calcein (D) versus GM1 content. The intensities of the intracellular Ca^{2+}-derived fluorescence and calcein fluorescence were expressed as fractional changes of untreated cells or of ionomycin-treated cells, respectively (taken as 100%). A variable number of cells (10-22) were analyzed for each experimental conditions in three different experiments. Error bars refer to S.D.

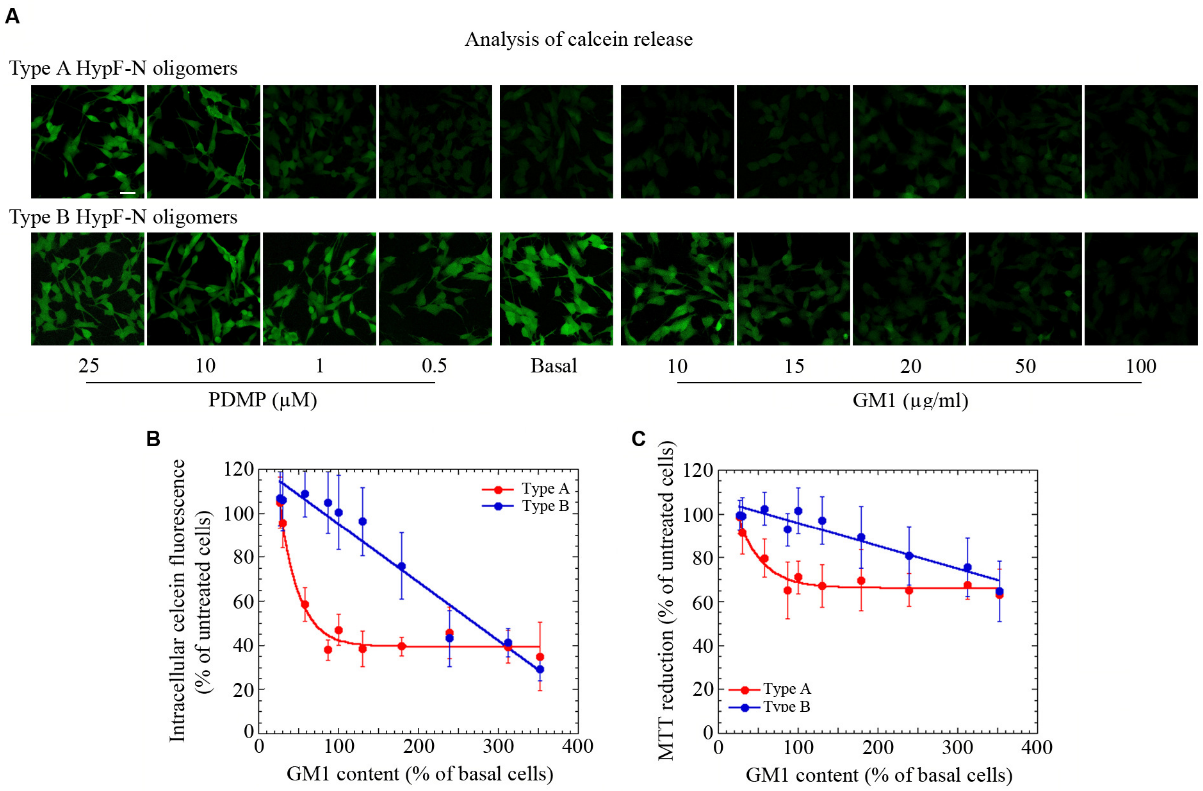

Figure S4. Membrane permeability and cytotoxicity in cells with different GM1 content exposed to different oligomers. (A) Representative confocal scanning microscopy images of basal, GM1-enriched (GM1) and GM1-depleted (PDMP) cells pre-loaded with calcein-AM and then exposed for 1 h to type A (upper images) or type B (lower images) HypF-N oligomers (12 μM monomer equivalent). Disruption of membrane integrity was monitored as a leakage of intracellular calcein (decrease of green fluorescence). (B,C) Plots showing the fluorescence associated with intracellular calcein fluorescence (B), or MTT reduction (C) versus GM1 content after treatment of the cells with HypF-N type A (red) and type B (blue) oligomers (12 μM monomer equivalent). The corresponding red and blue lines represent fits to hyperbolic and linear functions, respectively. Values of the intracellular calcein fluorescence were expressed as fractional changes of the effects on cells that had not been exposed to oligomers but pre-treated with same concentrations of GM1 or PDMP (taken as 100%). A variable number of cells ranging from 10 to 22 were analysed for each set of conditions, in three different experiments. Cell viability is expressed as the percentage reduction of MTT in treated cells compared with cells that had not been exposed to the oligomers but treated with the same concentrations of GM1 or PDMP. The values are means ± S.D. of four independent experiments carried out in quadruplicate.

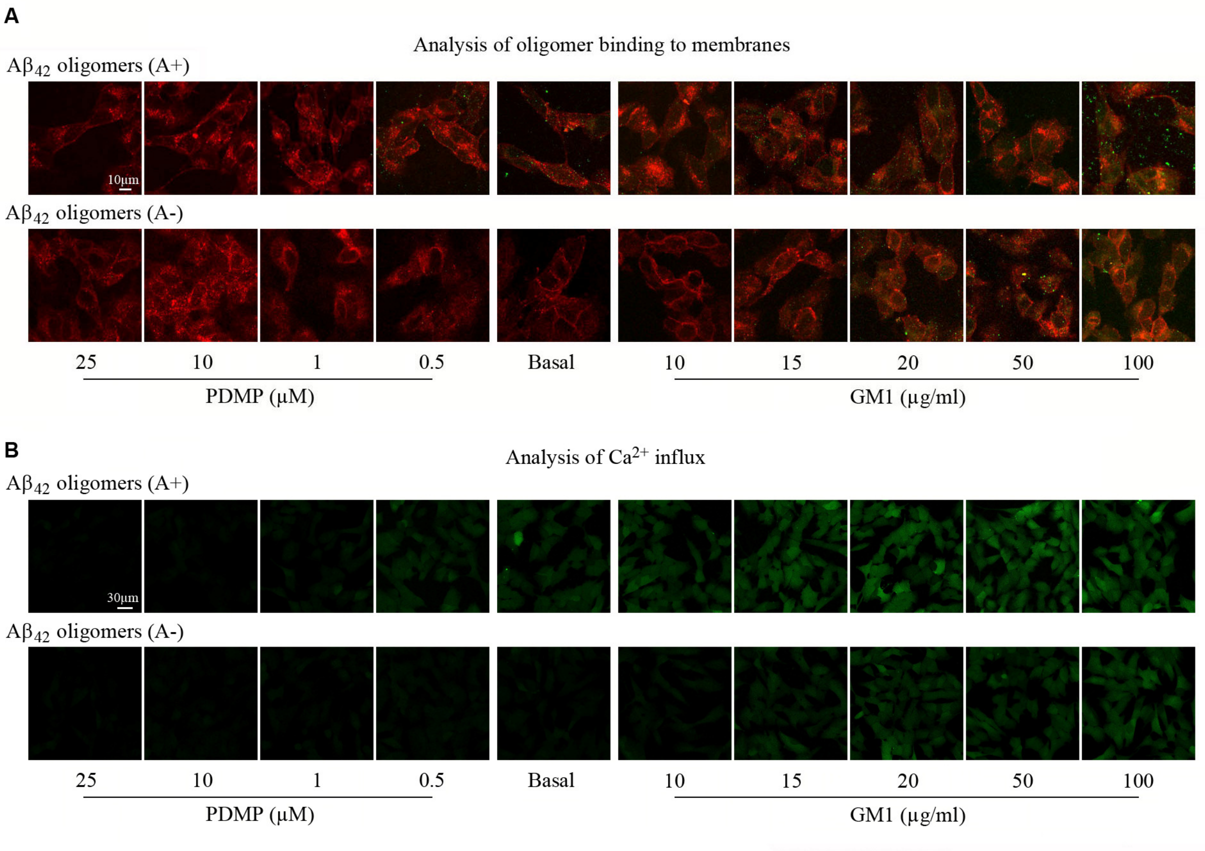

Figure S5. Analysis of the different recruitment of Aβ42 oligomers to the plasma membrane and the ensuing Ca^{2+} influx in cells with different GM1 contents. (A) Representative confocal scanning microscopy images of GM1-depleted (PDMP), basal and GM1-enriched (GM1) cells treated for 1 h with A+ (upper images) or A- (lower images) $A\beta_{42}$ oligomers at 10 μM concentration (monomer equivalent). In all images, red and green fluorescence indicates cell membranes and $A\beta_{42}$ oligomers, respectively. (B) Representative confocal scanning microscopy images of GM1-depleted (PDMP), basal and GM1-enriched (GM1) cells showing levels of intracellular free Ca^{2+} following treatment for 1 h with A+ (upper images) or A- (lower images) $A\beta_{42}$ oligomers at 10 μM concentration (monomer equivalent). In all images, the green fluorescence arises from Ca^{2+} binding to the intracellular Fluo3-AM probe.

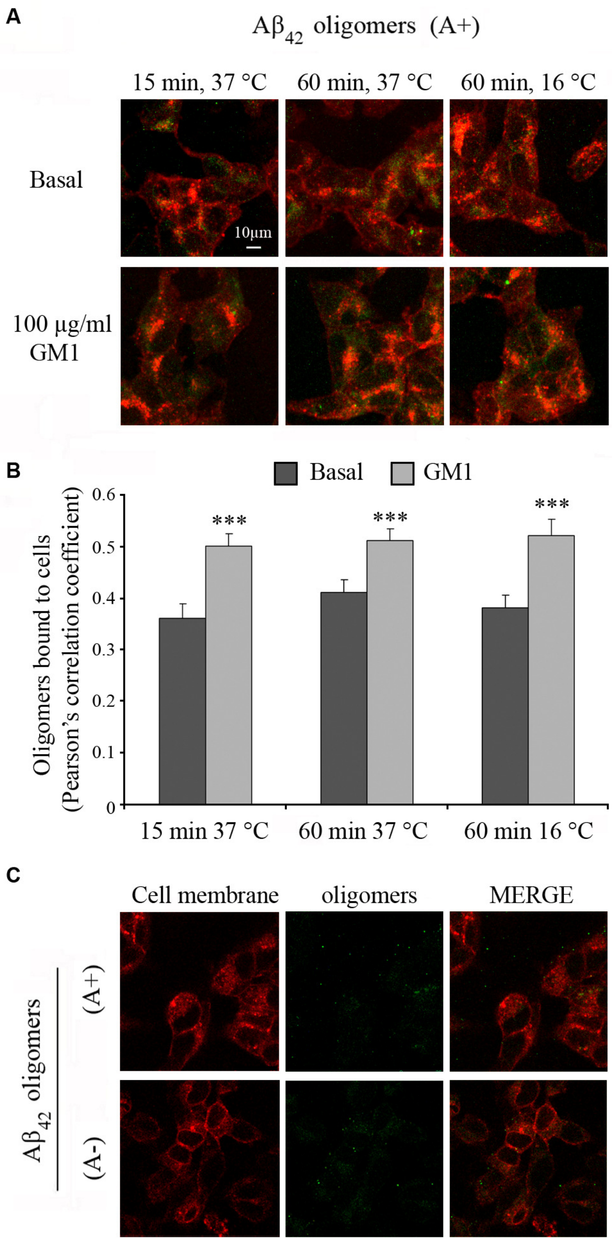

Figure S6. Binding of Aβ_{42} oligomers to cells with basal or increased GM1 content (A) Representative confocal scanning microscopy images of basal and GM1-enriched (100 µg/ml GM1) SH-SY5Y cells treated with A+ Aβ_{42} oligomers (10 µM, monomer equivalents) for 15 min at 37 °C, 60 min at 37 °C and 60 min at 16 °C. Red and green fluorescence emission is from cell membranes and Aβ_{42} oligomer (small dots), respectively. (B) Quantification analysis of the extent of oligomer binding to the cell membrane, represented as Pearson's correlation coefficient (PCC). (C) Representative confocal scanning microscopy images of the median sections of SH-SY5Y cells with increased GM1 content (100 µg/ml GM1) treated for 1 h with A+ or A– Aβ_{42} oligomers (10 µM, monomer equivalents). Variable numbers of cells (12-22) in three different experiments were analysed for each condition. Error bars refer to S.D.
